# OPPORTUNITIES FOR MHEALTH IN DEMENTIA CARE/CARE MANAGEMENT: LESSONS LEARNED FROM OTHER CLINICAL DISCIPLINES

**DOI:** 10.1093/geroni/igac059.686

**Published:** 2022-12-20

**Authors:** Hayley Belli

**Affiliations:** New York University School of Medicine, New York, New York, United States

## Abstract

The use of mHealth applications in dementia care/care management has recently become of increasing interest to patient stakeholders, clinicians, and researchers working in the field. However, there exists a rich literature of successful examples of the development, implementation, and efficacy of mHealth applications across a variety of health care disciplines. Thus there is high potential to take these lessons learned from other clinical fields and apply such successes to dementia care/care management. We will walk through several examples of mHealth tools used for managing hypertension, diabetes, migraines, and weight loss, while highlighting specific opportunities for dementia care/care management. Points of discussion will include optimizing the user-centered design process during the development phase of mHealth applications, increasing patient engagement with mHealth applications – especially among underrepresented individuals in research, promoting adherence to mHealth interventions via principles from behavioral economics, and scaling up mHealth interventions to test for efficacy at a population-level.

